# A Rare Presentation of Parvovirus Induced Pure Red Cell Aplasia in Elderly Male With Inclusion Body Myositis

**DOI:** 10.7759/cureus.12095

**Published:** 2020-12-15

**Authors:** Shobha Mandal, Ravindra Shah, Philip Lowry

**Affiliations:** 1 Internal Medicine, Guthrie Robert Packer Hospital, Sayre, USA; 2 Internal Medicine, Spinal Pain and Rehabilitation Medicine PC, New York, USA; 3 Hematology and Oncology, Guthrie Robert Packer Hospital, Sayre, USA

**Keywords:** pure red cell aplasia, parvovirus b-19, prca

## Abstract

Pure red cell aplasia (PRCA) is a rare condition leading to erythroid bone marrow failure. Parvovirus is one of the rare causes of PRCA in older adults. We present a 73-year-old man on high dose prednisone who presented with rapid functional decline and shortness of breath and was found to have normocytic normochromic anemia with low reticulocyte counts. On further workup, he was found to have elevated immunoglobulin M (IgM) titer of parvovirus B-19 antibody. The patient was managed with supportive care with blood transfusion, hydration and had improvement in his symptoms.

## Introduction

Pure red cell aplasia (PRCA) is a syndrome defined by normocytic normochromic anemia with severe reticulocytopenia (<0.5%) and marked reduction or absence of erythroid precursors from the bone marrow with normal leukocytes and thrombocytes count [1]. PRCA was initially described by Kaznelson in 1922 [2]. Parvovirus B-19 is a single-stranded DNA virus which is a rare cause of PRCA in adults but it can affect patients who are immunocompromised by leukemia, hemolytic anemia, acquired immunodeficiency syndrome, solid-organ transplant recipients. In our case patient was on immunosuppressive therapy because of inclusion body myositis. 

## Case presentation

**Figure 1:**
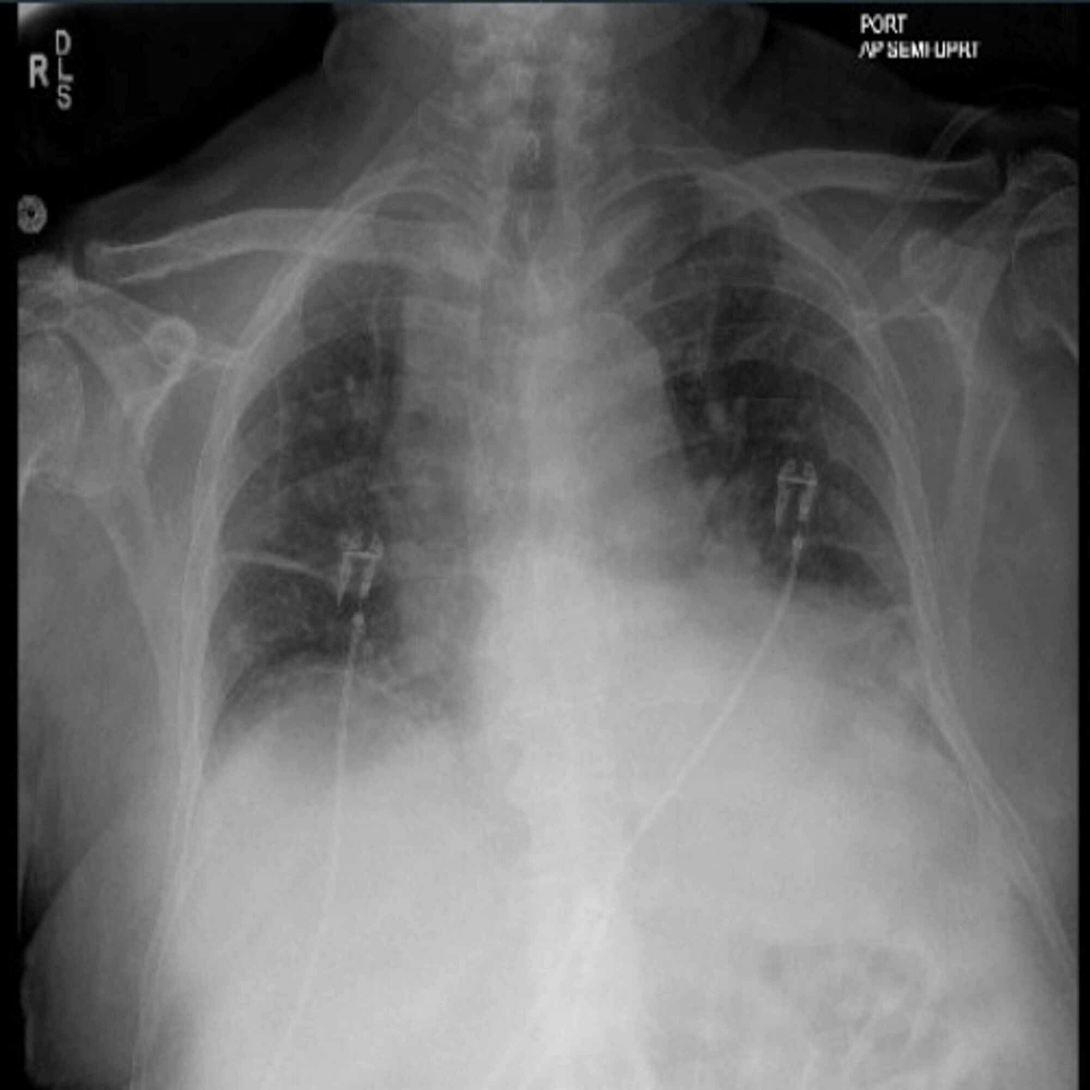
Anteroposterior chest X-ray showing findings suggestive of mild bilateral pleural effusion

A 73-year-old man with a history of biopsy-proven inclusion body myositis on high dose oral prednisone, intravenous immunoglobulin G (IVIG) and azathioprine therapy, hypertension, pulmonary embolism on rivaroxaban, congestive heart failure presented to the emergency department with worsening shortness of breath, and a rapid functional decline over the last one month. He denied any other complaints of chest pain, cough, blood in the stool, or recent blood loss. On physical exam, he had bilateral decreased breath sounds. A chest X-ray showed congestion with bilateral pleural effusion (Figure 1). 

Laboratory workup showed hemoglobin (Hb) of 9.8 g/dl (normal: 13.8-17.2 g/dl) with normal platelet, and white blood cell (WBC) counts. Other lab showed elevated N-terminal pro-brain natriuretic peptide (NT-proBNP) of 6000 pg/ml (normal <125 pg/mL). He was diagnosed with congestive heart failure exacerbation and was treated with intravenous (IV) Lasix®. The patient continued to have shortness of breath despite treatment with IV Lasix®. Hence he was also evaluated by neurology for his inclusion body myositis. The plan was made to start the patient on rituximab and wean him off the azathioprine and steroids.

He was also tested for hepatitis panel and QuantiFERON® gold, which came back negative. Further laboratory workup showed elevated ferritin levels, low total iron-binding capacity (TIBC), normal iron saturation, vitamin B12, and folate levels. The stool for the occult blood test was negative. During the course of hospitalization, the patient's hemoglobin dropped to 6.8 g/dl; hence he was evaluated by gastroenterology and underwent esophagogastroduodenoscopy, which was normal. Patient was treated with supportive care and further laboratory workup was done, which showed elevated lactate dehydrogenase (LDH) of 1052 U/L (normal: 140-280 U/L), haptoglobin 45 mg/dl (normal: 50-220 mg/dL), total bilirubin 1.8 mg/dl (normal: 0.1 to 1.2 mg/dL), reticulocyte 0.62% (normal: 0.5-1.5%) and reticulocyte count of 0.0167, which was suggestive of erythroid hypoplasia/aplasia. A peripheral blood smear was normal, with no schistocytes, spherocytes, or major abnormal red cell forms.

As the patient had a history of myositis, there was suspicion of muscular bleeds or retroperitoneal bleeds given the low hemoglobin with elevated LDH, low haptoglobin, and elevated bilirubin. The patient underwent computed tomography of the abdomen and pelvis, which was negative for any bleed. Our patient had normocytic normochromic anemia with low reticulocytes on the laboratory workup, and the duration of response after blood transfusion was short-lived; hence fundamental problems with erythropoiesis were found. Bone marrow involvement was less likely as the patient had intact white cells and platelet counts. The patient was tested for autoimmune conditions like systemic lupus erythematosus, rheumatoid arthritis, which came negative. Viral infections workup including HIV, hepatitis virus, and Epstein-Barr virus were negative, but parvovirus B-19 antibody immunoglobulin M (IgM) titer was elevated 1.2 IV (normal: <0.9 IV). He was managed with supportive care with blood transfusion. He received 5-6 units of packed red blood cell transfusions, and his hemoglobin remained stable in the range of 9-10 g/dl; hence the patient was discharged home with a follow-up appointment with a primary care physician in two weeks. On the follow-up visit, the patient reported improvement in his symptoms of shortness of breath. Repeat labs showed hemoglobin of 10 g/dl. 

## Discussion

PRCA is a rare erythroid bone marrow failure with normocytic normochromic anemia and reticulocytopenia with a normal count of white blood cells and platelets [3]. PRCA can be classified as congenital and acquired. Acquired PRCA can be further classified into primary and secondary. The congenital disorders usually manifest themselves early in life, and the congenital causes of PRCA are Diamond-Blackfan anemia, Fanconi anemia, and congenital dyserythropoietic anemias. Primary acquired PRCA is more commonly autoimmune in origin but can also be idiopathic. Secondary acquired PRCA may be associated with collagen vascular disease, autoimmune conditions such as systemic lupus erythematosus; lymphoproliferative disorders such as chronic lymphocytic leukemia, large granular lymphocyte leukemia, Hodgkin and non-Hodgkin lymphoma, Waldenstrom macroglobulinemia; infections, particularly B19 parvovirus; thymoma and other solid tumors; immunosuppressive drugs such tacrolimus, mycophenolate mofetil, azathioprine, cyclosporine corticosteroids; anti-HIV drugs, lamivudine, zidovudine; antibiotics such as linezolid, trimethoprim/ sulfamethoxazole; antiepileptic drugs like carbamazepine, valproic acid; antifungal drugs micafungin and several others drugs or toxic agents [3, 4]. Pregnancy and anti-erythropoietin (anti-EPO) antibodies are also associated with PRCA. Pregnancy-induced PRCA usually resolves after delivery [5]. 

Parvovirus is a small, nonenveloped, single-stranded DNA virus with tropism for the erythroid progenitor cells [6]. It is a member of the Parvoviridae family, well known to be pathogenic to humans [7]. Parvovirus is known to cause lytic destruction of the proerythroblasts leading to mild anemia in the immunocompetent person and children. It causes transient anemia associated with an erythematous rash called “fifth disease” or erythema infectiosum [8]. Occasionally, immunocompetent adults present with the symmetric polyarthropathy similar to rheumatoid arthritis. The tropism to erythroid progenitors in the bone marrow is well known to cause a transient aplastic crisis (TAC) in individuals with underlying hemolytic anemia and hydrops fetalis in intrauterine infections as viral growth can also be maintained in fetal liver cells [9]. In immunocompromised patients, the inability to generate an immune response leads to a state of persistent anemia, reticulocytopenia caused by lytic destruction of proerythroblasts giving rise to an acquired PRCA. However, it is very uncommon to have parvovirus infection in immunocompetent elderly [10].

On laboratory workup, patients with PRCA are found to have normochromic and normocytic anemia with absolute reticulocyte count less than 10,000/µL (reticulocyte percentage <0.5%) and normal white blood counts and platelet counts. Bone marrow biopsy is essential for the diagnosis of PRCA. In primary acquired PRCA, there is an absence of or near absence of erythroblasts from an otherwise normal marrow (<1% erythroblasts on the marrow differential count) with normal myeloid and megakaryocyte maturation and marrow cellularity.

In parvovirus infection, a few proerythroblasts and/or basophilic erythroblasts are seen on bone marrow biopsy, usually 5% of the differential count. Large proerythroblasts with vacuolated cytoplasm and pseudopodia (“giant pronormoblasts”) on bone marrow biopsy are suggestive of B19 parvovirus infection but are not diagnostic [11]. The presence of the anti-parvovirus B19 immunoglobulin M (IgM) and positive polymerase chain reaction (PCR) for a high load of parvovirus B19 on peripheral blood is the diagnostic test of choice. 

The treatment of PRCA is variable and is based on the causative agent. Any patient diagnosed with parvovirus induced PRCA should be managed initially with supportive care to keep the hemoglobin more than 7 gm/dl. The definitive treatment of diagnosed PRCA secondary to B19 parvovirus is IVIG. It is a highly specific and effective therapy. In the immune-competent host, 0.4 g/kg/day of IVIG is given for three days. In the immunocompromised host, generally, a higher dose of 1 g/kg/day is used for five days or longer. In patients, the prolonged duration of IVIG viral load was reduced to undetectable levels on repeated PCR testing [10].

## Conclusions

Parvovirus is a rare cause of pure red cell aplasia in immunocompetent adults. It is more common in children than in adults treated with immunosuppressive therapy. In any adult presenting with PRCA, physicians should always consider testing for parvovirus B19 and treat accordingly.
